# Unexpected High Blood Lead Levels in a Remote Indigenous Community in the Northeastern Peruvian Amazon

**DOI:** 10.3390/toxics13100826

**Published:** 2025-09-27

**Authors:** Pedro Mayor, Guillem Rius-Taberner, Gabriela M. Ulloa, Martí Orta-Martínez

**Affiliations:** 1 Departament de Sanitat i d’Anatomia Animals, Facultat de Veterinària, Universitat Autònoma de Barcelona (UAB), 08193 Barcelona, Spain; 2 Museo de Culturas Indígenas Amazónicas, Fundamazonia, Iquitos 16006, Peru; 3 ComFauna, Comunidad de Manejo de Fauna Silvestre en la Amazonía y en Latinoamérica, Iquitos 16006, Peru; 4 Department of Evolutionary Biology, Ecology and Environmental Sciences, University of Barcelona (UB), 08007 Barcelona, Spain; guillem.rius@isglobal.org (G.R.-T.); marti.orta@ub.edu (M.O.-M.); 5 Barcelona Institute for Global Health (ISGlobal), 08036 Barcelona, Spain; 6 Faculty of Medicine and Life Sciences, Universitat Pompeu Fabra (UPF), 08005 Barcelona, Spain; 7 Programa de Pós-Graduação em Saúde e Produção Animal na Amazônia, Universidade Federal Rural da Amazônia (UFRA), Belém CEP 66077-830, PA, Brazil; gulloau92@gmail.com; 8 Grupo de Enfermedades Infecciosas Re-Emergentes, Universidad Científica del Sur, Villa el Salvador, Lima 15067, Peru; 9 Institut de Recerca de la Biodiversitat (IRBio), University of Barcelona (UB), 08007 Barcelona, Spain

**Keywords:** lead poisoning, lead-based ammunition, drinking waters, subsistence hunting, Indigenous Peoples, Amazon, planetary health

## Abstract

Recent studies suggest that Pb-based ammunition could be an important route of Pb exposure for Indigenous Peoples in tropical rainforests. We analyzed blood lead levels (BLL) and isotopic signatures in 111 humans, 97 wild animals, 81 fish, and potential environmental Pb sources in an Indigenous community in the remote and well-preserved Peruvian Amazon with no history of industrial activity. Median BLL was 11.74 μg dL^−1^, with BLL ≥ 5 µg dL^−1^ in 95.8% children <12-yo and 94.5% adults. Pb concentrations in wild animals were 7.00 ± 22.40 mg kg^−1^ DW in liver, 0.06 ± 0.09 mg kg^−1^ DW in fish muscle tissues, 17.1 ± 10.8 mg kg^−1^ in soils and 3.4–3.8 mg L^−1^ in the main river, although 0.43-0.53 mg L^−1^ were the Pb levels in decanted water used for drinking and cooking. The similarity of isotopic signatures (^207/206^Pb and ^208/206^Pb) shows that the main Pb sources for humans are river waters (97.6%) and Pb-based ammunition (78.7%). Fish and wildlife act as Pb transporters from water, and wildlife act as Pb transporter from ammunition. Evidence of high human BLL in a remote, non-industrialized Amazonian area demonstrates the urgency of designing regional policies that include health prevention measures, focused on drinking water filtration systems and the use of non-toxic, Pb-free ammunitions.

## 1. Introduction

Lead (Pb) is one of the most persistent and widespread toxic metals globally, with the capacity to bioaccumulate across trophic levels and affect multiple organ systems [1]. Exposure to Pb is associated with neurodevelopmental impairment in children [2,3], as well as neurological and kidney damage, cardiac disease, lowered levels of fertility, and other health problems in adults [4,5]. No blood lead levels (BLL) are considered safe, and deleterious effects have been reported even at minimal exposure levels in both children and adults [3,6,7,8,9].

The global health burden attributable to Pb exposure is especially severe in Low- and Middle-Income Countries (LMIC), accounting for approximately 5.5 million deaths annually and contributing to 62.5% of cases of idiopathic developmental intellectual disability worldwide [10,11].

Nowadays, Pb contamination is typically associated with a regional history of industrialization, including mining, smelters, battery manufacturing, and recycling [12]. Thus, the occurrence of Pb in concentrations that pose a risk to humans and ecosystems in remote regions, such as tropical forests, has been widely assumed to be minimal due to few major anthropogenic putative sources.

However, despite the presumed low levels of anthropogenic disturbance in the Amazon, recent studies in remote Indigenous communities have reported high Pb levels in human blood—a geometric mean of 19.3 μg dL^−1^ [13]—and in wild game species—0.49 mg kg^−1^ wet weight [14], suggesting that Pb contamination may be more widespread in the Amazon than previously assumed. Finally, a causal relationship between human BLL and the consumption of wild game species has emerged throughout Pb isotopic analysis [13,14], and survey studies [15], identifying Pb-based ammunition as the final source of Pb exposure.

These findings uncovered significant and new health risks for Indigenous communities that rely on subsistence hunting and use Pb-based ammunition worldwide [14]. The introduction of Pb-based firearms and ammunition into the Amazon occurred in the early 20th century; however, the widespread use of shotguns for subsistence hunting began in the last decade of the century, primarily due to their high cost for Indigenous Peoples [16]. Within two decades, Pb pollution from shot ammunition may have emerged as a major environmental and public health concern even in the most remote and isolated parts of the Amazon.

The few existing studies on BLLs in the Amazon are based on small sample sizes or lack isotopic validation. Moreover, no comprehensive assessment of Pb contamination across human, animal, and environmental samples has been conducted in the region. Thus, this study aims to improve the understanding of Pb sources and exposure pathways of subsistence-based Indigenous Peoples in well-preserved forests in the Amazon. We present a cross-sectional, multi-compartment study of Pb levels and isotopic fingerprints of key biotic and abiotic elements in an Indigenous population in the Yavarí-Mirim River basin, an isolated, non-industrialized, and well-preserved area of the Peruvian Amazon. The analysis of Pb includes humans, wild game species, fish, and potential natural and anthropogenic sources (soil, water, ammunition, and fishing net weights). Despite logistical constraints, this baseline study aims to uncover potential pathways of exposure and provide critical insight into an emerging environmental health issue affecting Amazonian Indigenous communities.

## 2. Materials and Methods

### 2.1. Study Population

**Figure 1:**
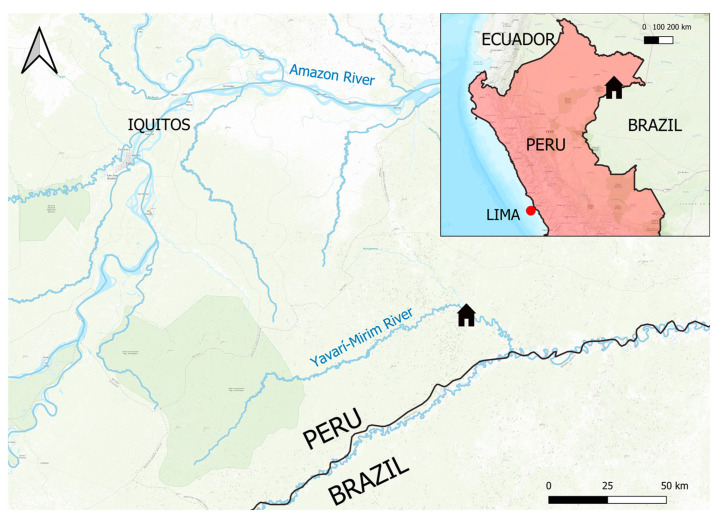
Location of the Yagua Indigenous community of Nueva Esperanza, the only settlement in the Yavari-Mirín River basin, northeastern Peruvian Amazon.

The Yagua Indigenous community of Nueva Esperanza (04°19′53″ S; 71°57′33″ W; UTC-5) is located in the Yavarí-Mirín River (YMR) basin in the Peruvian Amazon (Figure 1). The nearest urban settlement is Iquitos, 150 km away in a straight line. Due to the absence of a road network, access to Nueva Esperanza is limited to opportunistic river transport [17].

The local inhabitants of Nueva Esperanza (329 individuals in 46 households) rely on a subsistence-based economy. They depend on their territory for survival, engaging primarily in fishing, hunting, and slash-and-burn agriculture. Nueva Esperanza is the only community in the YMR basin, and there are no industrial activities throughout the area.

The area is characterised by continuous, well-preserved, and predominantly upland forest that hosts rich biodiversity [18]. Annual temperatures range from 22 to 36 °C, relative humidity between 80% and 100%, and annual rainfall between 1500 mm and 3000 mm.

### 2.2. Human Sampling

In February 2020, physicians performed a clinical examination and collected whole venous blood in vacuum tubes with EDTA K2 from 111 residents (36.5% of the total population). The sample included 66 (59.5%) women and 45 (40.5%) men, median age of 23 (Q1 = 10, Q3 = 34; range from 2 to 94 yo). The age categories were composed of 24 children <12 yo (21.6% of the sampled people and 19.4% of the total target population), 24 teenagers between 12 and 18 yo (21.6% and 43.6%, respectively), 52 adults between 19 and 49 yo (46.8% and 48.4%), and 11 old adults >50 yo (9.9% and 45.8%). Samples were transported in liquid nitrogen and subsequently stored at −70 °C until analysis.

### 2.3. Environmental Sampling

In 2019, a total of 12 soil samples and 14 samples of drinking water from different sources were collected (see Table 1 and Table 2 for the type and number of samples for each category). Three soil subsamples separated by at least 1.5 m were collected from the surface soil (between 0 and 20 cm deep) and were mixed and homogenized to constitute each soil sample. Soil samples were transported in plastic bags at room temperature and stored frozen in the laboratory.

Between 2010 and 2015, local hunters collected liver samples from 97 wild game specimens of 13 species, and, in 2015, fishermen collected the dorsal muscle from 81 fish specimens of 8 species. Wild animals and fish specimens were opportunistically collected by local inhabitants, taking advantage of their legal subsistence activities. Hunters and fishermen were previously trained to collect the liver from wild animals and whole fish specimens, and store them in a 4% (*v*/*v*) buffered formaldehyde solution. Biological samples were transported at room temperature in formaldehyde and placed in a freezer upon arrival at the laboratory.

Furthermore, four cartridges of the most frequently used ammunitions by local hunters (Armusa^®^, Bornaghi^®^, Drago-Cheddite^®^, and Saga^®^) and three ammunition pellets found by researchers during the handling of hunted animals were collected. Finally, two Pb rods used to prepare fishing nets were also collected.

### 2.4. Interviews for Risk Exposure

In February 2020, two types of face-to-face semi-structured questionnaires were administered to the local population to estimate Pb exposure risks (see Supplementary Table S1). Given the small size of the community, a convenience sampling approach with near-complete coverage was used. All households were invited to participate, and family heads from 34 households (73.9% of total households) agreed to respond to the family questionnaire to collect information on potential socio-cultural risk factors associated with Pb exposure pathways. For the individual survey, adult members from these households were approached, and 80 individuals (47.6% of the adult population; 34 men and 46 women) aged 37.2 ± 15.2 years (range 18–82 years) consented to participate. The response size for each question varied because some participants declined to answer sensitive questions.

Interviews were carried out by individuals unknown to the local population to reduce social desirability bias [19]. Prior to each interview, volunteers were informed of the purpose of the study to ensure comfort and transparency. Participation was entirely voluntary, and respondents were free to withdraw at any time. Confidentiality was assured, and collected data were anonymized to ensure that no identifying information could be disclosed.

During the periods January–April 2019 and September–December 2019, seven volunteer families participated in a dietary monitoring exercise, recording their consumption of animal protein and fish in their main meals. The total recording effort amounted to 513 days, 332 days during the first period and 191 days during the second.

No statistical association was established between risk exposure and BLL, due to the limited sample size with available information on both BLL and risk behaviors.

### 2.5. Laboratory Analysis

The scientific literature reports that formalin penetrates the outer perimeter of tissues up to 1 cm deep [20,21]; therefore, to reduce the risk of leaching and external contamination, the surface (about 1 cm) of the biological samples was removed using a ceramic knife [21]. Liver, muscle, and soil samples were freeze-dried and ground with a porcelain mortar and pestle. The soil samples were also sieved (0.6 mm).

An aliquot of each sample (0.1 g for liver and muscle samples, 0.25 g for soil samples) was digested with HNO_3_ in a microwave (Ultrawave, Milestone Srl, Bergamo, Italy) at 240 °C for 15 min for liver, at 90 °C for 24 h for muscle samples, and at 240 °C for 30 min for soil samples. Water samples (5 mL) were not digested but were brought to a pH of approximately 1 using concentrated HCl. Blood venous samples (0.2 g aliquot) were not digested but were diluted in 0.1% (*w*/*v*) EDTA, 2% (*v*/*v*) NH_3_, 4% (*v*/*v*) n-butanol and 0.1% (*v*/*v*) triton. Ammunition and fishing weight samples were treated with concentrated HNO_3_ for 48 h at room temperature. Digestion and dilution blanks were created simultaneously.

All samples were diluted with 1% HCl and then analyzed on a single quadrupole mass analyzer (7500ce, Agilent Technologies, Santa Clara, CA, USA) via inductively Coupled Plasma Mass Spectrometry (ICP-MS) to quantify Pb concentrations. According to the most dilute calibration standard, the lowest quantifiable concentrations are 0.25 mg kg^−1^ and 0.5 mg L^−1^. Pb concentration of the blanks was below the limit of detection (LOD). To measure analytical reproducibility (RSD = 15%), a laboratory-created reference standard was injected every 15 samples. By adding a Pb reference standard to some samples, precision and recovery (99%) were calculated at different concentrations. A concentric nebulizer and a double-pass propylene chamber were used for all ICP-MS injections.

BLLs and Pb concentrations are expressed using the median, the mean and, the standard deviation. Accuracy and recovery checks were performed using reference standard materials of (1) bovine liver NIST 1577c for liver samples; (2) dried, powdered soil NIST 2711 for use in the analysis of soils, sediments, or other materials of a similar matrix (National Institute of Standards Technology, US Department of Agriculture, Beltsville, MD, USA); and (3) human blood BCR-635 for blood samples (Institute for Reference Materials and Measurements, Joint Research Centre, Geel, Belgium). Recovery (*n* = 5) was 95.27 ± 1.70% for liver samples, 103.08 ± 0.004% (mean ± S.D.) for blood, and 75.63 ± 0.02% for soils. Pb concentration values are expressed in relation to the sample’s dry weight (DW).

To rule out the contribution of Pb from formaldehyde and/or Pb leaching from biological samples, Pb concentrations in formaldehyde were measured before and after sample preservation. Two samples of the initial formaldehyde and five samples from different drums were analyzed from inside the individual plastic bags. Samples of formaldehyde solution (0.1 g aliquot) were digested with HNO_3_, HCl and HF in a microwave (Ultrawave, Milestone Srl, Italy) at 240 °C for 15 min and analyzed. The mean Pb concentration was 0.013 ± 0.000 μg mL^−1^ in the initial formaldehyde, and 0.014 ± 0.008 μg mL^−1^ in the formaldehyde after four years. Consequently, the probability of Pb leaching, external contamination and cross-contamination between stored samples was considered to be negligible.

Signals for ^204^Pb, ^206^Pb, ^207^Pb and ^208^Pb isotopes were determined using quadrupole ICP-MS. The NIST 981 standard Pb reference material was used every five samples to correct for mass discrimination. Only samples with Pb concentrations above the limit of quantification were used, minimizing error in isotopic determination. Pb isotopic composition was expressed as relative concentration ratios, including ^207^Pb/^206^Pb (SD ± 0.0035) and ^208^Pb/^206^Pb (SD ± 0.0094). Due to isobaric interference from ^204^Hg, isotopic ratios involving the isotope ^204^Pb were not used.

### 2.6. Data Analysis

Based on the cumulative total of 513 recorded days of diet, the following indicators were estimated for each participating household: meals per day and daily consumption of fish, domestic and game animals (body mass, in percentage and in grams). Meat and fish consumption (in grams) was estimated after adjusting for carcass yield, according to Eslava [22], and Bardales-García et al. [23], respectively.

Given the positively skewed distribution of BLL reported, we modeled the data using a generalized linear model (GLM) with a Gamma distribution and a log link function. The GLM included age (continuous) and sex (categorical: male/female) as predictors. We also tested for an interaction between age and sex to evaluate whether the effect of age on BLL differed by sex. Statistical analyses were performed using R-Studio version 2024.09.0 Build 375 (RStudio, Inc., Vienna, Austria) and Deducer JRG version 1,7–9, 2003–2011 RoSuDa, Univ. Augsburg). Differences with a probability value of 0.05 or lower (*p* < 0.05) were considered significant.

To determine the sources of BLLs, we compared the ratios of Pb isotopes (^207/206^Pb and ^208/206^Pb) from blood samples of local people with samples identified as potential sources of Pb contamination, including wild game and fish specimens, soils, drinking waters, Pb-based ammunition, and Pb rods that local inhabitants use to prepare fishing nets. Plots of ^207/206^Pb and ^208/206^Pb isotope ratios were used to pinpoint the source of Pb. The mean and SD of the ^207/206^Pb, ^208/206^Pb, and ^207/208^Pb ratios were used to estimate the square area of the Pb isotopic fingerprint of the putative sources at a 95% and 99% confidence interval (mean ± 1.96×SD and mean ± 2.58×SD, respectively). The Pb isotopic similarity between sample categories was calculated based on the percentage of samples within the area of influence of the isotopic fingerprint of each putative Pb source.

### 2.7. Ethical Authorizations

The aspects of the study involving humans were performed with the approval of the Ethics Committee of the Universidad Peruana Cayetano Heredia (No. 29-3-19 and 270-10-19), the Autonomous University of Barcelona (No. CEEAH 4829), and the Hospital Clínic de Barcelona (HCB/2019/1107). The study was presented to the local authorities of Nueva Esperanza, as well as regional and local authorities (No. 267-2019-GRL-DRSL/30.09.01). The research protocols for the sampling of NHPs were approved by the Peruvian Forest and Wildlife Service (041–2007-DGGFS-DGEFFS, 0350–2012-DGFFS-DGEFFS, 258–2019-MINAGRI-SERFOR-DGGSPFFS) and the Institutional Animal Use Ethics Committee of the Universidad Peruana Cayetano Heredia (No. 102142). Biological samples from wildlife were exported with the approval of the Peruvian Forestry and Wildlife Service (00605-CITES-Perú and 003106-CITES-Perú, 001309-MINAGRI-DGFFS and 003005-SERFOR).

The objectives of the study were presented in community meetings. Prior to participation in the study, written informed consent was obtained from participants ≥18 years, personal verbal consent and informed written paternal consent from participants between ≥7 and <18 years, and informed written paternal consent from participants <7 years was obtained.

## 3. Results

### 3.1. Human Lead Levels

The median BLL was 11.74 μg dL^−1^ (Q1 = 7.63, Q3 = 15.59; *n* = 111), ranging from 3.45 to 38.71 μg dL^−1^ (geometric mean BLL was 12.96 ± 7.17 μg dL^−1^); of the total participants, 94.59% had BLL ≥ 5 µg dL^−1^, 60.36% ≥ 10 µg dL^−1^ and 12.6% ≥ 20 µg dL^−1^.

The median BLL for children (<12 yo) was 9.49 (Q1 = 7.18, Q3 = 12.29; *n* = 24) (geometric mean 11.26 ± 7.56 μg dL^−1^); of the total children, 95.8% had BLL ≥ 5 µg dL^−1^, 41.7% ≥ 10 µg dL^−1^ and 8.3% ≥ 20 µg dL^−1^. For adults (≥12 yo), the median BLL was 12.33 (Q1 = 7.96; Q3 = 17.10; *n* = 96) (geometric mean 13.42 ± 7.04 μg dL^−1^); 94.5% had BLL ≥ 5 µg dL^−1^, 62.1% ≥ 10 µg dL^−1^, and 13.8% ≥ 20 µg dL^−1^.

**Figure 2:**
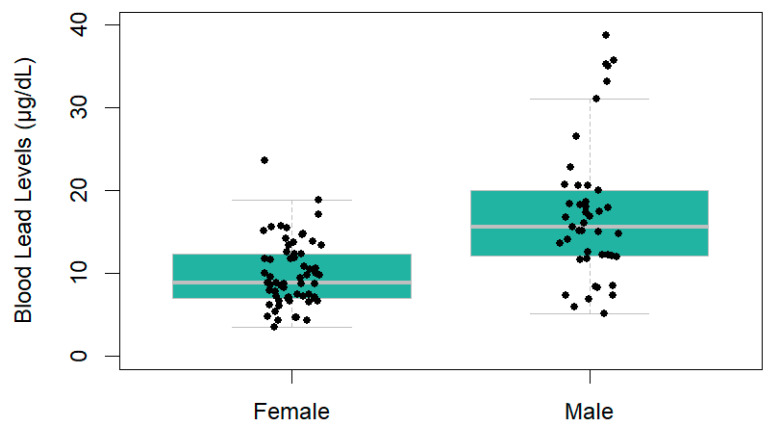
Blood Pb levels in local men (*n* = 45) and women (*n* = 66) of Nueva Esperanza in the Yavari-Mirín River.

BLL were significantly higher in men than in women (β = 0.570, *p* = 0.0006); however, age was not a statistically significant predictor (β = 0.0051, *p* = 0.139). In men, the median BLL was 15.56 μg dL^−1^ (Q1 = 12.06; Q3 = 20.03; *n* = 45) (geometric mean 17.27 ± 8.40 µg dL^−1^), while for women it was 8.84 μg dL^−1^ (Q1 = 7.03; Q3 = 12.51; *n* = 66) (geometric mean 10.06 ± 4.24 μg dL^−1^) (Figure 2). 

### 3.2. Environmental Lead Levels

The median Pb concentration in wild game livers was 2.24 (Q1 = 1.62, Q3 = 3.96 mg kg^−1^ DW; *n* = 97) (geometric mean 7.00 ± 22.40 mg kg^−1^ DW or 1.70 ± 5.52 mg kg^−1^ WW), ranging from 0.46 to 199.44 mg kg^−1^ DW. In the dorsal muscle of fish, the median Pb concentration was 0.0269 mg kg^−1^ (Q1 = 0.0149, Q3 = 0.0707 mg kg^−1^ DW; *n* = 81) (geometric mean 0.061 ± 0.088 mg kg^−1^ DW or 0.014 ± 0.019 mg kg^−1^ WW), ranging from 0.0007 to 0.083 mg kg^−1^ DW.

In soils, the geometric mean Pb concentration was 17.11 ± 10.81 mg kg^−1^ (*n* = 12; Table 1), with the highest levels detected in soils collected within the community (30.11 ± 5.88 mg kg^−1^, *n* = 6).

In drinking waters, the geometric mean Pb concentration was 1.03 ± 1.24 mg L^−1^ (*n* = 14; Table 2), with the highest levels detected in samples collected from the YMR (3.4 and 3.8 mg L^−1^). Notably, water samples from the YMR that were allowed to settle in households showed lower Pb levels, ranging from 0.43 to 0.53 mg L^−1^. Further laboratory sedimentation of the same water samples using centrifugation at 3000 rpm resulted in superficial water Pb concentrations below 0.04 mg L^−1^.

### 3.3. Isotopic Fingerprint

**Figure 3:**
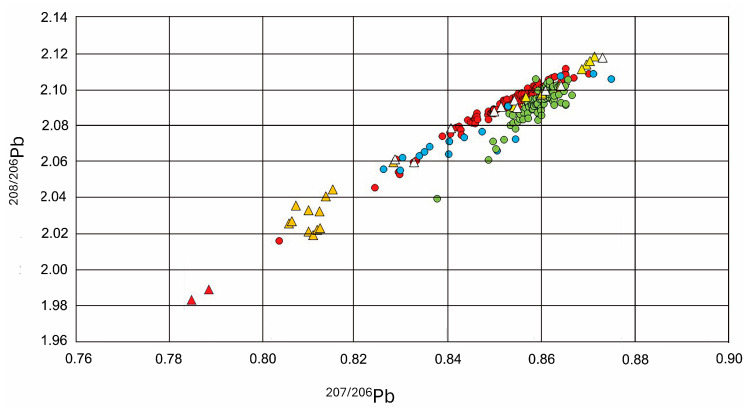
Isotopic fingerprint (^207/206^Pb and ^208/206^Pb ratios) in biological and environmental samples collected around the community of Nueva Esperanza in the Yavari-Mirín River. Dots represent biological samples, including human blood samples (red dots, *n* = 111), liver from wild game species (green dots, *n* = 97) and dorsal muscle from fish (blue dots, *n* = 81), and triangles represents environmental samples, including soils (orange triangles, *n* = 6), drinking waters (white triangles, *n* = 14), ammunition (yellow triangles, *n* = 7), and Pb rods used to prepare fishing nets(red triangles, *n* = 2).

The isotopic fingerprint ratios (^207/206^Pb and ^208/206^Pb) in human blood samples showed high similarity with those of YMR waters (97.6%) and fish samples (99.7%), and substantial similarity with those of ammunition (78.7%) and wild hunted specimens (68.2%) (Figure 3).

The isotopic signature observed in fish closely matched that of drinking waters (94.7%) and showed moderate overlap with ammunition (45.6%). The isotopic fingerprint observed in wild hunted specimens was highly similar to that of drinking waters (97.6%) and ammunition (96.6%).

In contrast, there was little or no overlap between the isotopic signatures of soils or fishing net weights and any of the biological matrices analyzed. Moreover, a low overlap (13.3%) between the isotopic signatures of Pb in drinking water and soils was reported (Figure 3). No significant variation was observed in the isotopic fingerprint of human blood samples with respect to age and sex.

### 3.4. Dietary Patterns

Over a cumulative total of 513 recorded dietary days, we recorded an average of 2.19 ± 0.44 meals per day in each participant household. The most consumed sources of animal protein were, in decreasing order: fish (50.9%, 261/513), wild meat (37.8%, 194/513), meat from domestic animals—poultry—(9.9%, 51/513), and canned meat (1.4%, 7/513). The average daily animal consumption per inhabitant was 189 ± 139 g of fish and 158 ± 30 g of wild animals (body mass). This weight includes organs such as bones, scales, and other horny structures. After adjusting for edible yield, daily meat intake was 73 ± 53 g (26.7%) of fish meat, 139 ± 27 g (51.1%) of wild meat, and 60 ± 68 g (22.2%) of domestic meat per inhabitant.

Overall, 88.8% of animal protein originated from subsistence fishing and hunting. Moreover, 90.0% of fish and 67.4% of wild animals were consumed within the household, and a smaller portion was shared with neighbors (6.8% of fish meat and 20.2% of wild meat). All wild animals (100.0%, 108/108) were hunted by adult men, while 69.3% of the fish (158/228) were captured by adult women. No hunting or fishing activities were reported by children under 18 years.

### 3.5. Sociocultural Risk Factors

Each household was composed of 5.9 ± 2.6 persons, including 2.6 ± 14.3 children (<12 yo), 1.4 ± 1.5 youths (13–18 yo), 1.6 ± 1.1 adults (19–59 yo), and 0.3 ± 0.7 old adults (>60 yo).

Overall, 59.3% of adults reported that they usually fish, whilst a smaller proportion hunted (36.0%). In each household, 1.12 hunters go hunting once a week. Shotgun was reported as the predominant way of hunting (87.5%), although 12.5% of hunters also employ dogs during hunting activities (Supplementary Table S1).

The most frequently consumed protein was fish, 6.7 times per week, whereas wild meat was consumed 2.6 times per week. Wild meat was usually hunted (70.6%) by someone in the same family, and 50% would share the meat with neighbours or family. The type of ammunition used in the community is caliber 16, with a pellet diameter between 0.61 cm and 0.91 cm. When eating, they often (83.3%) reported finding shotgun pellets in cooked meat, although 78.8% of participants reported that shotgun pellets were easy to remove from the meat, and 91.2% reported they removed them before cooking.

Most respondents (88.2%) indicated that shotgun Pb pellets were repurposed as weights for fishing nets and rods. To shape them, 20.6% (18.7% females and 22.2% males) reported chewing the pellets. Those who reported biting Pb pellets prepare their fishing nets 20.1 days a year (*n* = 34), with the frequency being higher in men than in women (35.0 versus 7.7 days/year, respectively). Complementary comments from interviewees reported the alternative use of special Pb bars for fishing equipment, and the introduction of hammers to shape and fix the Pb in the fishing nets and rods.

Respondents reported collecting drinking water from a combination of sources. The most common was rainwater (82.7%), followed by water from the YMR (54.3%) and smaller tributary rivers (24.9%). Despite not being the most frequently consumed drinking water, all surveyed families reported consuming water from the YMR. These same sources were also used for cooking, with only 38.3% of respondents usually boiling water before drinking it. No use of lead-glazed ceramic cookware was reported in any of the surveyed households.

Finally, smoking was reported by 29.8% of individuals, with an average of 8.3 cigarettes per person-week. Additionally, 54.9% of households had at least one family member who smokes occasionally.

No statistical association was established between risk exposure and BLL, due to the low sample size with concomitant information on BLL and risk behaviors.

## 4. Discussion

In the absence of significant anthropogenic sources of Pb in the vicinity of this site, this study offers a unique opportunity to assess Pb exposure routes in the particular socioeconomic context of subsistence hunting and fishing in Indigenous Amazonian communities.

### 4.1. High BLLs in Indigenous People

We report very high BLLs among Yagua Indigenous People living in a remote and isolated area in the Peruvian Amazon, where there are no industrial sources of Pb. Although no BLL is considered safe, since 2015, the US National Institute for Occupational Safety and Health has established a threshold of 5 μg dL^−1^ to define elevated BLL [24]. The frequencies of inhabitants with BLL above this threshold were 95.8% for children <12-yo and 94.5% for adults.

Neurodevelopmental, cardiovascular, renal, and reproductive effects have been observed at BLL 1–2 µg dL^−1^ [25,26,27,28,29]. In children, Pb exposure can cause irreversible neurodevelopmental effects, leading to cognitive and learning impairment, shorter attention spans, and disruptive behavior [30,31]. Fetuses and young children are particularly vulnerable to the effects of Pb because they absorb ingested Pb more efficiently, have limited ability to excrete it, and have rapidly developing nervous systems [25,31,32,33,34].

The values observed in the present study are higher than those reported for industrial areas in high-income countries and in urban areas from LMIC when leaded gasoline was permitted. In metropolitan areas of South America, average BLLs were 9.9 µg dL^−1^ for children and 3.4 µg dL^−1^ for women in 1998 in Lima, Peru [35], and 5.5 µg dL^−1^ for children in Cartagena, Colombia [36]. Among children aged ≤5 years, our measured BLLs are approximately five times higher than values reported in Europe (2.6 μg dL^−1^) between 1999 and 2007 when leaded gasoline was still permitted [37]. Values were similar to those of the U.S. population in 1976–1980 (12.8 μg dL^−1^) and ten times higher than values reported in the U.S (0.84–1.65 µg dL^−1^) between 1999 and 2014 [38,39].

There are very few studies on BLLs from Amazonian Indigenous Peoples. Our results double the values observed in Indigenous Peoples from the northern Peruvian Amazon in areas affected by oil extraction activities (4.9 µg dL^−1^) [40]. Similarly, our values largely exceed those reported around the Peruvian Amarakaeri Communal Reserve in Madre de Dios (5.7 µg dL^−1^), an area more accessible from urban centers and affected by illegal mining activities [15]. In contrast, our results are similar to BLLs reported in other isolated areas of the Amazon, such as in the lower Tapajós River basin, Brazilian Amazon (13.1 µg dL^−1^) [41] and in the Camopi Indigenous People of French Guiana (19.3 ± 8.4 µg dL^−1^) [13].

### 4.2. Sources and Exposure Routes for Pb

In the absence of confounding Pb sources such as industrial activities (such as oil and gas extraction), leaded paint or leaded gasoline, the combined analysis of the isotopic fingerprints and Pb levels indicate that the main exposure routes of Pb for the Yagua Indigenous People are drinking water, and, to a lesser extent, hunting ammunition. While drinking water is an important but probably local source of Pb, Pb-based ammunition is a much more important source of Pb for global Indigenous and traditional Peoples in rural forests.

#### 4.2.1. Lead Contribution Through Water

Although Pb is a problem in drinking water in many parts of the world, there is still a significant lack of awareness of the extent of the problem in LMIC. Our findings—both the elevated Pb levels and the isotopic analyses—indicate that drinking water from the YMR constitutes a major pathway of Pb exposure. In the Amazon, Indigenous communities typically consume two main types of water for drinking and cooking: rainwater and river water. Amazonian rivers have a seasonal hydrological regime with significant water level changes that cause extensive fluvial erosion processes due to river dynamics [42], where Pb could be a naturally occurring component [1].

Based on evidence of attributed health impacts, regulatory agencies in the United States, Canada and the European Union have established maximum allowable concentrations in water ranging from 0.005 to 0.015 mg L^−1^ [25,29,43,44]. The concentrations detected in the YMR water (3.6 mg L^−1^) are between 250 and 750 times higher than the maximum allowable concentrations established in drinking water. However, the drinking water consumed by local households is decanted water, which has much reduced Pb level (0.43–0.53 mg L^−1^, 32 times higher than the maximum permissible levels). Laboratory centrifugation further reduces Pb concentrations to <0.4 mg L^−1^.

Although Pb concentrations in water are generally low, average Pb levels in the YMR waters were much higher than the 0.015 mg L^−1^ found in untreated water samples from the Amazon River in Colombia [45], or even the 0.013–0.21 mg L^−1^ in natural water associated with mining activity also in Colombia [46], or 0.698–0.750 mg L^−1^ in small rivers affected by discharge from the brick industry, coal mining and wastewater from nearby communities of Sogamoso, Colombia [47].

In the absence of nearby industrial sources or other human communities, the moderate similarity (58.3%) in the Pb isotopic fingerprint between the river water and the local soils around the Indigenous community of Nueva Esperanza suggests that the YMR is mobilizing geogenic Pb with a different isotopic composition. The soils analyzed—including agricultural soils, natural saltpeter soils, and community soils—originate from high-altitude forests. Due to their distinct origin and composition, these upland soils show a relatively distinctive isotopic signature that differs from river sediments.

#### 4.2.2. Lead Contribution Through Hunting Ammunition

The isotopic fingerprint analysis revealed substantial overlap between humans, wild hunted animals, and Pb-based ammunition, supporting the hypothesis that dietary intake of wild meat hunted with Pb-based ammunition constitutes a primary source of Pb exposure in the population. These findings back previous evidence based on BLL, dietary questionnaires, and indirect exposure assessments, which indicated a context-dependent relationship between the consumption of wild meat hunted with Pb-based ammunition and elevated BLLs in Amazonian populations [15,40,48].

The only previous study that has studied Pb isotopic fingerprints in Indigenous Peoples in the Amazon identified manioc and hunting ammunition as the major sources of Pb. However, it is worth mentioning that they did not analyze drinking water [13]. Similarly, a study conducted in Madre de Dios that did not use isotopic analysis to track human exposure to Pb found that elevated BLLs were associated with wild meat consumption, particularly when consumed in large quantities [15]. Similar results have also been documented in Global North contexts [49,50]. However, the BLLs reported in the present study are more than twice those observed in Madre de Dios. This discrepancy may be attributed to a greater dependence on wild meat as a primary protein source in the YMR basin, likely driven by the well-preserved populations of wildlife and limited access to domestic animals [48], as observed in local dietary patterns.

Differences in Pb exposure due to strongly gender-segregated occupations appear to explain the higher BLLs found in men compared to women and children. In the studied community, men spend more time in the forest hunting wildlife and logging, which increases wild meat consumption [51] and exposes them to Pb dust when guns are fired [52]. In addition, when men are away from the community, women often increase their fishing efforts to compensate for the reduced availability of wild meat. These gender-segregated BLLs consistently coincide with the literature in rural Amazonian populations [15,40]. Another potential occupational exposure to Pb in the study site is the reuse of ammunition scraps to manufacture fishing sinkers. This activity has also been reported as one main risk factor for higher BLLs in other Amazonian communities [41,53].

Pb levels in the studied wildlife livers (7.00 mg kg^−1^ DW or 1.70 mg kg^−1^ WW) are extremely high. According to the EU Commission Regulation 1881/2006, 0.5 mg kg^−1^ WW is the maximum permissible limit of Pb for foodstuffs, and 0.1 mg kg^−1^ WW for meat for human consumption. Our findings show that 52.6% and 99.0% of wildlife liver samples exceeded the maximum permissible limit of Pb for foodstuffs or meat for human consumption, respectively.

Our reported levels far exceed those reported in studies conducted on deer livers (0.27 mg kg^−1^ WW) and wild boar (0.60 mg kg^−1^ WW) from industrialized countries, where values typically range from 0.05 to 2.61 mg kg^−1^ [54,55,56]. These values are much higher than those reported in bovine liver in different areas of Colombia (0.49–0.62 mg/kg) [57]. This latter comparison is illustrative, as offal intake by Indigenous People is highly relevant to their food security.

Given the high isotopic similarity of the wildlife–human (68%) and wildlife–ammunition (97%) isotopic fingerprints, our results strongly indicate that wild meat is an important exposure route of Pb for Indigenous Peoples, driven by the use of Pb-based ammunition. These findings are consistent with previous studies indicating that wild meat is a significant source of Pb exposure among subsistence-hunting Indigenous Peoples in the Amazon [14,15].

Wild meat is a vital source of nutrition and income for millions of Indigenous Peoples and other rural communities in the Amazon and across the Global South [58]. Subsistence hunting relies on Pb-based ammunition, which is widely used in at least 62 countries affecting 150 million people [59]. Our findings highlight the significant global public health risks associated with this practice for global Indigenous Peoples [14,60].

#### 4.2.3. Lead Contribution Through Alternative Uses of Shot Pellets

The use of shot pellets as a practical, versatile complement to fishing nets and rods is a frequent behavior among Amazonian Indigenous Peoples and has already been reported as a risk behavior for Pb exposure [45,50]. In our study, 34 (20.6%) respondents (18.7% females and 22.2% males) reported biting pellets for this purpose 20.1 days a year (35.0 for men versus 7.7 days/year for women). Therefore, this should be considered a high-risk behavior. Nevertheless, important changes have been reported in the community in recent years: (1) the use of Pb bars instead of shot pellets to manufacture fishing weights and (2) the use of hammers to reduce pellet-biting behavior. Unfortunately, we did not quantify the frequency of these behavioral changes. It is worth mentioning that there is a complete divergence between the Pb isotopic fingerprint of fishing weights and that of lead ammunition.

#### 4.2.4. Lead Contribution Through Fish

According to the EU Commission Regulation 1881/2006, 0.3 mg kg^−1^ WW is the maximum permissible limit of Pb for fish meat for human consumption. The low concentrations of Pb found in fish (0.06 mg kg^−1^ DW or 0.014 mg kg^−1^ WW in muscle) resulted in no fish sample exceeding the maximum permissible limit of Pb. These low levels are consistent with the results of the limited studies available from South America (0.01 to 0.05 mg kg^−1^ WW [61,62]). However, some studies from Colombia have reported higher values in fish muscle, including 0.66–2.03 μg/g DW in the Magdalena River [63], 0.45 μg/g in the Chicamocha River [64], and 1.30 mg/kg in the Gulf of Urabá [65].

Wild meat intake was almost double that of fish (139 and 73 g per person per day, respectively). Given the relatively low Pb concentrations in fish, its contribution to overall Pb exposure appears negligible compared to wild meat.

The high similarity of the isotopic signatures of wildlife waters (98%) and fish waters (100%) shows that, as in humans, terrestrial and aquatic fauna consume river waters and act as a route of exposure to Pb from these waters. The low concentrations of Pb observed in fish, despite the high Pb levels detected in undecanted water and their isotopic similarity, remain unexplained. Further research, including controlled experimental studies, is needed to elucidate the pathways and mechanisms of Pb uptake and bioaccumulation in riverine fish.

#### 4.2.5. Lead Contribution Through Soils

All soils analyzed were sampled in the upland forest and included mineral licks and agricultural and community soils. All soils showed Pb concentrations (between 6.4 and 36.1 mg kg^−1^) below the reference values set by the US Environmental Protection Agency (EPA) for residential land use, with investigation recommended at 200 mg kg^−1^ or 100 mg kg^−1^ in areas with multiple Pb exposure sources [66]. Our findings are consistent with the Pb levels reported in manioc plantations of the Wayãpi Indigenous People in southeastern French Guiana (2.89 and 49 mg kg^−1^) [13]. Similar concentrations have been reported along the Tapajós River in Brazil (10.6–25.1 mg kg^−1^), where elevated Pb in manioc flour has been associated with local geology and soil composition [67]. In our study area, the isotopic analysis suggests a limited contribution of BLL from soil-derived Pb, potentially indicating a minor role of locally cultivated cassava and other crops in overall exposure, although this remains speculative.

Finally, while atmospheric deposition of anthropogenic Pb is a recognized pathway of contamination in remote ecosystems [68], our isotopic analysis suggests that this source is unlikely to have significantly contributed to Pb levels in local soils. The isotopic signatures of known atmospheric Pb sources in South America [69,70] differ from those observed in this study area [14].

### 4.3. Limitations

This study represents one of the most comprehensive assessments of Pb exposure for Indigenous Peoples, combining biological, environmental, isotopic, and sociocultural data into a unified, community-based research framework. However, this study has several limitations that should be considered when interpreting the findings. First, the cross-sectional design limits the ability to assess temporal variations in Pb exposure. Given that Pb has a biological half-life of approximately 30 days in blood before redistributing to soft tissues, bones, teeth, and hair [71], longitudinal studies provide greater insight into exposure dynamics and possible seasonal or behavioral fluctuations.

Second, the study was conducted in a single Indigenous community, which restricts the generalizability of the findings to other populations with different environmental, dietary, and cultural characteristics. Nevertheless, many of the subsistence practices observed, such as the use of Pb-based ammunition, are widespread throughout the Amazon and other tropical forest regions, suggesting that Pb-based ammunition might be a very relevant source for Pb poisoning. By contrast, the geological composition of the headwaters of the YMR basin might be specific to this area.

Third, although the combined biomarker, isotopic, and ethnographic evidence supports the contribution of contaminated water and wild game to Pb exposure, other potential sources, such as locally cultivated crops, were not analyzed. Previous studies have reported elevated Pb concentrations in cassava consumed by Indigenous populations in the Amazon [41]. Additionally, although reported tobacco use was low, the potential contribution of smoking to BLLs was not assessed [71].

Despite these limitations, the findings of this study provide novel and robust evidence of Pb exposure in an isolated Indigenous population in the absence of extractive activities or industries. To our knowledge, this is the first study to scientifically demonstrate the role of Pb-based ammunition and untreated river water as primary exposure pathways in the Amazon. These findings underscore the urgent need for context-sensitive public health strategies and regulatory interventions tailored to the realities of subsistence-based Indigenous communities.

## 5. Conclusions

Through a One Health interdisciplinary and community-based approach, this study revealed alarmingly high levels of Pb exposure in a remote Indigenous community of the Peruvian Amazon. Despite the absence of industrial activities, the main sources of Pb identified were river water and wild meat hunted with Pb-based ammunition, an integral part of daily life and subsistence in the region.

There is a large body of scientific literature on the toxic effects of Pb from ammunition on both wildlife and human health [60]. Effective, non-toxic Pb-free alternatives are available at market prices comparable to those of Pb-based ammunition, and regulatory options through legislation are straightforward [72,73,74]. However, vested political and economic interests seem to be significant impediments to the phase-out of Pb ammunition [60]. Furthermore, until now, almost no literature has addressed Pb poisoning for Indigenous Peoples, the largest hunter population in the world, and most efforts to ban or restrict the use of Pb-based ammunition have taken place in industrialized countries. Therefore, urgent progress is needed to phase out Pb ammunition in Indigenous contexts without negatively impacting subsistence hunting, whether in terms of participation, harvest levels, or economics [61].

Until alternative ammunition options are made available to Indigenous Peoples, a way to minimize the risk of exposure could be by removing the bullets and the surrounding meat. However, this mitigation strategy may not be as effective, as up to 30 cm of tissue surrounding the bullet’s trajectory is exposed to Pb due to bullet fragmentation [75,76]. Notably, most of the surveyed population reported finding pellets in cooked meat, although 91% said they usually remove them before cooking. However, it is not common in the region to remove the meat surrounding the bullet path. Another urgent measure to consider is to eliminate the reuse of shot pellets as weights in fishing nets and rods, a frequent practice among Amazonian Indigenous Peoples and other rural communities.

Our findings also highlight the urgency to consider action plans to reduce the consumption of river water with high Pb content. Mitigation strategies should prioritise the use of alternative water sources with lower Pb levels, such as rainwater. In this context, our study observed a notable reduction in Pb concentrations in water samples subjected to simple water decantation treatment in the tanks that families use to store water. These results suggest that more effective sedimentation or filtration techniques may substantially reduce Pb exposure from drinking water. However, further research and intervention studies are needed to identify the most effective and context-appropriate strategies in tropical forest environments [77].

These findings highlight an urgent and often overlooked public health issue affecting Indigenous and rural populations across the Amazon and beyond. Simple, culturally adapted interventions, such as improved water treatment and access to Pb-free ammunition, are essential to reduce exposure and protect vulnerable communities that remain disconnected from basic environmental health protections. Alongside bans on Pb ammunition in EU and other industrialized countries, restrictions on its use are urgently needed in LMIC in order to guarantee public health for Indigenous Peoples.

## Figures and Tables

**Table 1:** Lead (Pb) concentrations (in mg kg^−1^ DW) in 12 soil samples collected from different land-use areas around the community of Nueva Esperanza in the Yavari-Mirín River basin. Samples include subsistence hunting grounds, locations within the community, and agricultural plots.

Sample	Description	Code	Pb
Community	Soil samples collected from various locations within the community	S1	36.11
		S2	24.43
		S3	25.76
		S4	34.16
Agricultural Lands	Soil samples from agricultural plots	S5	14.35
		S6	10.14
Hunting hotspots	Soil samples from sites frequently used for subsistence hunting (e.g., mineral salt licks)	S7	6.37
		S8	6.93
		S9	11.95
		S10	20.5
		S11	7.04
		S12	7.63

**Table 2:** Lead (Pb) concentrations in 14 drinking water samples (in mg L^−1^) collected around the community of Nueva Esperanza in the Yavari-Mirín River (YMR) basin. Samples include different treatments of rainwater, main river YMR water, and water from a small nearby river.

Sample	Code	Treatment	Description	Pb
YMR	W1	Untreated	River water used directly for drinking	3.85
	W2	Untreated	River water used directly for drinking	3.4
	W3	Treated	River water left to settle before use	0.53
	W4	Treated	River water left to settle before use	0.43
	W5	Treated	River water centrifuged in the laboratory	<0.04
	W6	Treated	River water that is boiled before use	0.93
	W7	Treated	River water that is boiled before use	0.82
Small nearby river	W8	Untreated	Stream water collected directly	0.15
	W9	Treated	Sedimented stream sample	0.07
Rainwater	W10	Untreated	Collected from rooftops using metal gutters from different houses	1.66
	W11	Untreated	Collected from rooftops using metal gutters from different houses	0.88
	W12	Treated	Rainwater left to settle before use	0.21
	W13	Treated	Rainwater left to settle before use	0.17
	W14	Treated	Rainwater that is boiled before use	0.24

## Data Availability

The data presented in this study are available on request from the corresponding author. The data are not publicly available because it includes confidential information from people who participated in the study, even if they are anonymous.

## Supplementary Materials

The following supporting information can be downloaded at: https://www.mdpi.com/article/10.3390/toxics13100826/s1, Supplementary Table S1. Sociocultural characteristics of the study population related to Pb exposure in Nueva Esperanza on the Yavari-Mirín River.
