# Supplementary material for: Unexpected High Blood Lead Levels in a Remote Indigenous Community in the Northeastern Peruvian Amazon
Source: Toxics. 2025 Sep 27;13(10):826. doi: 10.3390/toxics13100826 (PMC12567903; doi:10.3390/toxics13100826)
Supplement: Supplementary file 1 [file toxics-13-00826-s001.zip › toxics-3848079-supplementary.pdf]

Supplementary Table S1. Sociocultural characteristics of the study population related to Pb exposure in Nueva Esperanza on the Yavari-Mirín River.

**Questions**	**Results**	**N**
Main activities in the forest	Fishing, 59.3%; Agriculture, 57.0%; Hunting, 36.0%; Logging, 23.3%; Fruit extraction, 20.9%	80
Hunters per household	1.12 ± 0.64 (CI 95% 0.89-1.35; range from 0 to 2)	34
Hunting frequency	1.00 ± 1.50 times per week (CI 95% 0.00-1.54; range from 0 to 7)	34
Hunting technique	Firearms, 87.5%; Dogs, 12.5%	24
Are pellets also used to prepare fishing nets?	Yes, 88.2% (CI 95% 76.1-99.6)	34
Do you use to bite lead-pellets to prepare your fishing nets?	Yes, 20.6% (CI 95% 6.5-35.9)	34
Did you observe people handling pellets?	Yes, 47.1% (CI 95% 29.4-64.7)	34
Do you think that handling pellets may be a health concern?	Yes, 38.3% (21.0-55.4%); No, 52.9%; No response, 8.8%	34
Do you think that biting pellets may be a health concern?	Yes, 64.7% (CI 95% 47.8-81.6); No, 32.4%; No response, 2.9%	34
Is it easy to remove all pellets from wild meat?	Yes, 78.8%	34
Do you usually remove the pellets from wild meat before cooking?	Yes, 91.2% (81.1-101.2)	34
How often do you find pellets in wild meat after being cooked?	Always, 17.6%; Frequently, 2.9%; Sometimes, 61.8%; Rarely, 5.9%; Never, 11.8%	34
Do you usually find pellets in wild meat before cooking?	Yes, 88.2% (CI 95% 76.8-99.6)	34

Frequency of wild meat consumption?	2.58 ± 1.85 times per week (CI 95% 1.92-3.23; range from 0 to 7)	34
Frequency of fish consumption?	6.71 ± 1.07 times per week (CI 95% 6.34-7.00; range from 4 to 7)	34
How do you get wild meat?	Hunting, 70.6%; Purchase (50%, 17/34); Ggift, 50.0%	34
Type of water consumed in the community?	Rain, 82.7%; Main river, 54.3%; Small rivers, 25.9%	80
Do you use to boil the water before drinking?	Yes 38.3% (CI 95% 210-55.4)	34
Type of water consumed in the forest?	Small rivers, 80.6%; Main river, 25.8%; Rain, 16.1%	31
Type of water for cooking?	Rain, 70.6%; Main river, 49.0%; Small rivers, 29.4%	34
Containers storing the consumed water?	Plastic deposits, 94.8%; Plastic tanks, 13.0%	77
Consumption of tobacco?	Yes, 29.8% (CI 95% 20.4-39.2)	80
Years smoking cigarettes?	17.03 ± 8.47 years (CI 95% 13.98-20.09; range from 0.1 to 34)	28
Number of cigarettes per week?	8.34 ± 17.66 (CI 95% 2.62-14.07; range from 0 to 100)	28
Does anyone smoke at home?	Yes, 54.9% (CI 95% 42.3-66.2)	71
